# Emerging and Innovative Technologies for the Sanitization of Fresh Produce: Advances, Mechanisms, and Applications for Enhancing Food Safety and Quality

**DOI:** 10.3390/foods14111924

**Published:** 2025-05-28

**Authors:** Yuqiao Jin, Achyut Adhikari

**Affiliations:** School of Nutrition and Food Sciences, Louisiana State University, Baton Rouge, LA 70803, USA; yjin@agcenter.lsu.edu

**Keywords:** combined sanitization method, ozone, ultraviolet light, cold plasma, pulsed light, ultrasound, microbubbles and nanobubbles, electrolyzed water, high-pressure processing, chlorine dioxide gas

## Abstract

The consumption of fresh produce has significantly increased in recent years, contributing to improved diets through the provision of essential nutrients, vitamins, and fiber. However, there has been a rise in foodborne illness outbreaks linked to fruits and vegetables, often caused by pathogens such as *Escherichia coli* O157:H7, *Salmonella* spp., and *Listeria monocytogenes*. These outbreaks have led to severe health consequences, including illnesses, hospitalizations, and even deaths. Once produce is contaminated by foodborne pathogens, these pathogens are difficult to eliminate. Traditional decontamination methods, such as water washes and chlorine-based sanitizers, have been widely used to address these microbial concerns. However, these methods may not be effective against pathogens in crevices or biofilms on the surface of produce, and their effectiveness varies depending on the type of produce and pathogens. Moreover, the chemicals used may raise health and environmental concerns. As a result, novel technologies for pathogen inactivation are gaining attention. These include ozone, ultraviolet light, cold plasma, pulsed light, ultrasound, microbubbles, nanobubbles, electrolyzed water, high-pressure processing, chlorine dioxide gas, and among others. This paper reviews a range of emerging and innovative technologies for the sanitization of fresh produce. The mechanisms, advancements, and practical applications of these technologies are examined with a focus on enhancing food safety and preserving produce quality. These innovative methods provide new opportunities for both research and industry to develop practical, affordable, and safe solutions for maintaining produce safety and quality. Recent studies highlight the effectiveness of combining methods, showing that using multiple sanitization techniques can significantly improve pathogen inactivation on fresh produce. For example, more than 5 log reductions of *Listeria innocua* and *E. coli* on avocado, watermelon, and mushroom can be achieved with the combined application of pulsed light and malic acid in previous research. In this review, we recommend the application of combined sanitization methods, emphasizing that integrating multiple techniques can provide a more effective and comprehensive approach to pathogen inactivation. This combined-method strategy has become a promising and innovative trend in the ongoing efforts to improve produce safety and quality.

## 1. Introduction

The consumption of freshly picked produce in the U.S. has dramatically increased in recent years [1,2]. The produce serves as a good source of nutrients, vitamins, and fiber for a healthy diet [2]. However, the outbreak of foodborne illness in fruits and vegetables has increased with the increasing availability of produce worldwide [3,4]. The Centers for Disease Control and Prevention (CDC) has reported recent outbreaks of *Escherichia coli*, *Salmonella* spp., and *Listeria monocytogenes* linked to fresh produce such as mushrooms, basil, peaches, nectarines, plums, cucumbers, cantaloupes, and packaged salads [5,6,7,8,9,10,11,12]. These outbreaks have caused severe illness, hospitalization, and even death across the states [5,6,7,8,9,10,11,12]. For example, *Salmonella* infections linked to cantaloupes led to 407 illnesses, 158 hospitalizations, and 6 deaths across 44 states in the U. S. [9].

Microbial contamination is the most common cause of produce deterioration, either contaminated at the farm or during or post-harvesting [13]. As fresh produce is often eaten raw without processing step, there is a need to control microbial outbreaks on fresh produce before consumption [1,2]. Traditional technologies and common approaches use water with or without sanitizers to wash fruits and vegetables [4]. Water washing can remove contamination from produce but may transfer pathogens around and cause re-contamination [2,14,15]. Chlorine-based sanitizer is widely applied in the produce industry to control microbiological safety for its relatively low cost and ease of use [2,16]. Chlorine is a strong oxidant that can interact with cellular components and affect metabolic processes [17,18]. Usually, washing solutions containing 20–200 ppm free chlorine can eliminate pathogens between 1 and 3 log_10_ CFU/g [19]. For example, 30 s dip treatment with hypochlorite solution containing equal or higher than 50 mg/L free chlorine resulted in 1.9–2.8 log_10_ CFU/g reduction of *E. coli* from lettuce leaves [20]. However, chlorine can react with soluble organic compounds to form trihalomethanes which is deleterious for human dietary safety and may cause environmental pollution [21]. The chlorine water may not be effective if the pathogen is inside the organic matter on the produce or in the crevices [22,23,24,25]. Other chemical methods such as chlorine dioxide and peroxyacetic acid have been studied to produce sanitation. Aqueous chlorine dioxide treatment was significantly (*p* < 0.05) more effective than water in reducing *Salmonella* spp. (2.14 vs. 1.44 log_10_ CFU/cm^2^ within 20 min), *L. monocytogenes* (1.98 vs. 0.49 log_10_ CFU/cm^2^ after 30 min), and *E. coli* O157:H7 (2.1 log_10_ CFU/cm^2^ after 20 min) on sweet potatoes [26]. In another study, washing with 30 mg/L chlorine dioxide for 2 min yielded a 90% reduction of *S. enterica* on the leaf surface of iceberg lettuce [27]. Although chlorine dioxide has limited sanitation efficacy, it prevents cross-contamination of iceberg lettuce leaves even at high organic loads in the washing water [27]. Singh et al. (2018) reported that peroxyacetic acid at 100 mg/L had better microbial inactivation of *E. coli* O157:H7, *Salmonella* Typhimurium DT104, and *L. monocytogenes* on lemon, tomato, cantaloupe, blueberry than bleach at 100 mg/L of free chlorine, 2% lactic acid, and DI water in the automated produce washer for 5 min [28]. However, the sanitizing efficacy on coarse surfaces such as cantaloupe and lettuce was lower than on smooth surfaces such as lemon, tomato, and blueberry, as the bacteria may be harbored in the crevices [28].

It is difficult to sanitize fresh produce under different conditions. Besides the types of sanitizers, previous studies have shown that the efficacy of microbial decontamination is dependent on many factors such as surface characteristics of produce and bacteria properties [1,28,29]. Pathogens could penetrate inside if the surface of the produce is injured [15]. A rough surface increases bacteria and biofilm attachment and protects them against shear forces [30,31,32]. Wash with or without sanitizer may not remove all pathogens as they could be in the cut edges and pores of the produce. The bacteria property can also affect the sanitization results. It was reported that *L. monocytogenes* was more resistant than *E. coli* and *S. enterica* in chlorine dioxide water washing on iceberg lettuce [27]. Also, bacteria can adhere to the surface and produce extracellular polymeric substances to form a biofilm [31]. The biofilm protects the communities of bacteria from environmental stresses such as desiccation, acids, and antimicrobials [19,33,34,35,36]. Destroying biofilms with chemical sanitizers is challenging [37,38].

Fruits and vegetables are usually not cooked before being eaten, which poses significant food safety concerns to the public if the produce is contaminated by pathogens. Conventional decontamination processes utilize a huge amount of water and chemicals. Concerns of traditional sanitizers with respect to the formation of by-products and chemical residues have been rising in the past years [39]. As there is a demand for the enhanced availability of fresh produce with high safety and quality, increasing attention has been focused on novel technologies to eliminate pathogens. Novel technologies in the sanitation of fresh produce include ozone, ultraviolet, cold plasma, pulsed light, ultrasound, microbubbles, nanobubbles, electrolyzed water, high-pressure processing, chlorine dioxide gas, and combined methods. The U. S. Food and Drug Administration (FDA) approved novel methods such as ozone, UV-C light, pulsed light, high-pressure processing, and chlorine dioxide gas. With the regulatory acceptance, various techniques have been developed to remove and inactivate pathogens in fruits and vegetables. This paper reviews a range of emerging and innovative technologies for the sanitization of both whole and fresh-cut fruits and vegetables. The mechanisms, advancements, practical applications, and previous research in literature have been discussed.

## 2. Novel Technologies

### 2.1. Ozone

Mechanism. Ozone is triatomic oxygen which can naturally occur as a form of oxygen [40,41]. It can be generated naturally by ultraviolet irradiation from sunshine and commercially by UV lights (at 185 nm) or corona discharge [21,42,43]. The relative molecular mass of ozone is 48 g/mol, the density is 2.14 g/L at 0 °C and 101.3 kPa, the boiling point is −112 °C, and the oxidative potential is 2.07 eV [40,44]. The utility of ozone as a sanitizing agent to inactivate microorganisms depends on the strong oxidation effect on the cell membranes and causing membrane damage and leakage [45]. It can decompose and generate hydroxyl free radicals •OH which are the main reactive oxidizing agents to inactivate microorganisms [46,47,48,49]. The FDA accepted ozone as an antimicrobial agent in the treatment, storage, and processing of foods (21 CFR173.368) [50]. Both aqueous and gaseous-phased ozone can be used in direct contact with foods, including fresh produce [50]. The efficacy of ozone depends on both intrinsic parameters (water quality, air quality, treatment concentration, treatment time) and extrinsic parameters (type of produce, surface characteristics, surface area, microbial strains, microbial load) [41,51,52].

Application (aqueous ozone). The ozonated water is generated through ozone gas pumped into water and used in fruit and vegetable washing [42]. Unlike chlorine-based washing systems, ozonated water is free of chemicals [53]. Examples of ozone applications are summarized in Table 1. There were 4.8 and 2.9 log_10_ CFU/g reduction of *E. coli* O157:H7 on raspberries and strawberries, respectively, after 64 min aqueous ozone treatment at 20 °C [54]. No significant color difference was found between the treated fruit samples and untreated samples [54]. Aqueous ozone treatments at 1.4 mg/L were assessed for the impact on the shelf-life of fresh-cut apples [55]. Results showed that 5 and 10 min treatments reduced total bacterial counts by 1.83 and 2.13 log_10_ CFU/g, respectively, compared to the control after 12 days of storage at 4 °C [55]. Liu et al. (2020) washed the fresh-cut cabbage with aqueous ozone (1.4 mg/L) for 10 min and stored the cabbage for 12 days at 4 °C [56]. The aqueous ozone treatment inhibited the growth rate of aerobic bacteria and delayed the decline of sensory for the treated cabbage [56]. Aqueous ozone can also destroy pesticides and chemical residues [13,57]. For example, applying the aqueous ozone (1.4 mg/L) for 5 min on the fresh-cut cabbage significantly removed trichlorfon, omethoate, chlorpyrifos, dichlorvos, and methomyl compared with the control [56].

Application (gaseous ozone). The gaseous ozone can be pumped into the air space of the postharvest storage facility and storage unit [42]. It is a strong sanitation and fumigation agent which can control bacteria, yeast, mold, and insects on product surfaces during storage to extend shelf-life [58]. Commodities such as strawberries and mushrooms which are susceptible to water can be treated with gaseous ozone instead of aqueous ozone [13]. Uniform exposure could lead to an effective reduction of pathogens on the produce surface in a controlled condition [42]. It was reported that the 6% wt/wt gaseous ozone in oxygen in a laboratory scale reduced 2.1 log_10_ CFU/g of *Salmonella* spp. on fresh strawberries after a 30 min treatment [59]. Another study utilized a lab-scale ozone generator and applied the continuous ozone gas (5.00% wt/wt) at a flow rate of 0.34 m^3^/h on raspberries and strawberries for 64 min [60]. There were 2.6 and 1.8 log_10_ CFU/g reduction of *E. coli* O157:H7 on raspberries and strawberries, respectively, without any observed color change on the products after ozone [60]. However, uniform contact exposure may not be achieved in packed cartons or bins [42]. Caution should be taken when applying gaseous ozone in commercial storage rooms or containers as the infections are usually within or beneath the produce surface [42]. Gaseous ozone can eliminate undesirable flavor from bacteria and slow down the ripening process in postharvest treatment and cold storage [43,61]. Fruits and vegetables release ethylene gas, which is a ripening hormone to accelerate the ripening process [62]. With chemical reaction, ozone serves in removing ethylene to prolong the storage life [63]. It was reported that ozone at a concentration of 0.04 μL/L was effective in removing ethylene from the atmosphere of the pear and apple storage room [61].

Combined method (aqueous ozone). Utilizing a combined-method approach has emerged as a new trend for sanitizing fresh produce. For example, Sun et al. (2022) applied a combined treatment of ultrasound (28 kHz), chlorine (free chlorine at 10 ppm), and aqueous ozone (1 ppm) to fresh-cut lettuce [64]. The combined treatment of ultrasound–chlorine (40 s) followed by 1.0 ppm aqueous ozone (60 s) did not negatively affect sensory quality attributes such as color, flavor, and crispness, nor did it alter total color difference [64]. Additionally, the Browning-related enzyme activities were significantly reduced [64]. This treatment also resulted in significantly lower populations of *S. typhimurium*, *E. coli* O157:H7, aerobic mesophilic bacteria, and molds and yeasts over 7 days of storage [64].

Combined method (gaseous ozone). A pilot-scale system combing vacuum cooling and ozone gas at sanitizing level (ozone at 1.5 g/kg gas-mix) inactivated 1.8 log_10_ CFU/g of *E. coli* O157:H7 on spinach without apparent quality damage after a holding time of 30 min at the holding pressure of 10 psig [65]. Pyatkovskyy et al. (2017) combined the application of gaseous ozone and liquid sanitizer spray to effectively inactivate *E. coli* O157:H7 on baby spinach [66]. In this study, the most effective combination was the initial spray of Pro-San L (containing 0.66% citric acid and 0.036% sodium dodecyl sulfate), followed by vacuum cooling and ozonation (at a pressure of 68.9 kPa) [66]. This combined method reduced 3.9 log_10_ CFU/g of *E. coli* O157:H7 on baby spinach [66]. The long-term use of a combined liquid-gaseous sanitizer treatment showed a better visual appearance of fresh produce compared to a single application of either gaseous or liquid sanitizers alone [66].

Advantages. Ozone has been known as an effective sanitizer to treat fresh produce. It has fast reaction kinetics with high oxidation potential, which is 1.5 times stronger than chlorine and 3000 times stronger than hypochlorous acid [42,43]. It can rapidly attach the bacterial cell walls and thick-walled spores of plant pathogens with a practical and safe concentration [42]. Desired inactivation results are achieved with low concentration and short contact time, compared with weaker oxidizing agents [67]. With a short half-life, released ozone will not stay in the environment for a long time and form harmful compounds [41,67]. Ozone does not leave residues nor form by-products such as chlorinated hydrocarbons and trihalomethanes [41,42,67]. It is suitable for fresh-cut or processed fruits and for treated products with an organic claim [68]. The strong odor of ozone can be sensed by humans at a low concentration of 0.01 μL/L to prevent harmful situations when it is dispersed in the environment accidentally [43].

Disadvantages. The high cost of ozone generators limits their application in the agriculture industry [41]. The ozone should be generated onsite and immediately used [69]. Each ozone generator unit should have the Environmental Protection Agency (EPA) establishment number and a device registration number [42]. Aqueous ozone is not stable and is more susceptible to dirt and organic materials compared with gaseous ozone [13]. Although ozone does not leave any residue, oxidized products may form deleterious properties [42]. Excessive exposure to the ozone may cause injury to the tissue of produce and reduce the storage and sensory life [41,42]. Like all oxidizing agents, ozone may affect human health when exposed at a relatively high concentration and long duration [13,42,70]. Severe symptoms such as water eyes, tightness in the chest, shortness of breath, and headaches were shown in individuals who were exposed to a high concentration, and even death over the concentration of 4 μL/L [13,42]. The Federal Occupation Safety and Health Administration (OSHA) sets the threshold of 0.1 μL/L for 8 h, and 0.3 μL/L for 15 min for healthy individuals [13]. Measures such as proper personnel protective equipment, monitoring systems, and exhausting systems should be taken to prevent any unnecessary exposure [13]. Handling and applications of ozone should be conducted under Good Manufacturing Practices (GMP) [42]. High concentrations or prolonged exposure to ozone during the treatment of fresh produce can lead to quality degradation, including alterations in texture, color, sensory, and nutritional content [13,41,71]. To mitigate these adverse effects, ozone can be combined with other hurdle technologies such as ultrasound and microbubbles [41,64,71,72].

**Table 1:** Examples of ozone-based microbial inactivation in fruits and vegetables.

Treatment	Commodity	Treatment Parameters (Dose, Time, Temperature, etc.)	Microorganism	Reduction (log_10_ CFU/g)	Ref.
Aqueous ozone	Raspberry	8.9 mg/L 64 min, 20 °C	*E. coli* O157:H7	4.8	[54]
			*Salmonella* spp.	4.4	[54]
	Strawberry	8.9 mg/L, 64 min, 20 °C	*E. coli* O157:H7	2.9	[54]
			*Salmonella* spp.	3.3	[54]
	Fresh-cut apple	1.4 mg/L, 10 min	Total bacteria	0.87	[55]
Gaseous ozone	Strawberry	6% (wt/wt), 30 min	*Salmonella* spp.	2.1	[59]
			*Enterococcus faecium*	1.5	[59]
	Raspberry	5%(wt/wt), 64 min	*E. coli* O157:H7	2.6	[60]
			*Salmonella* spp.	1.6	[60]
	Strawberry	5%(wt/wt), 64 min	*E. coli* O157:H7	1.8	[60]
			*Salmonella* spp.	0.9	[60]
Ultrasound, chlorine, and ozone (aqueous)	Lettuce leaves	Ultrasound (28 kHz)-chlorine (free chlorine 10 ppm), 40 s + aqueous ozone (1 ppm), 60 s	*E. coli* O157:H7	>2	[64]
			*S. typhimurium*	>2	[64]
Vacuum ozone (gaseous)	Spinach	1.5 g/kg, 30 min, at 10 psig	*E. coli* O157:H7	1.8	[65]
Pressured ozone (gaseous)	Raspberry	5%(wt/wt), 64 min, at 83 kPa	*E. coli* O157:H7	2.8	[60]
			*Salmonella* spp.	2.0	[60]
	Strawberry	5%(wt/wt), 64 min, at 83 kPa	*E. coli* O157:H7	2.3	[60]
			*Salmonella* spp.	2.2	[60]
Continuous ozone, pressurized ozone	Raspberry	5%(wt/wt), 64 min + 83 kPa, 64 min	*E. coli* O157:H7	3.8	[60]
			*Salmonella* spp.	3.6	[60]
	Strawberry	5%(wt/wt), 64 min + 83 kPa, 64 min	*E. coli* O157:H7	2.9	[60]
			*Salmonella* spp.	2.6	[60]
Sanitizer and ozone (gaseous)	Baby spinach	0.66% citric acid, 0.036% sodium dodecyl sulfate, spray 32 times + vacuum cooling (4 °C) + gaseous ozone (1.5 g/m^3^), 30 min	*E. coli* O157:H7	3.9	[66]

### 2.2. Ultraviolet (UV)

Mechanism. Ultraviolet (UV) light is part of the electromagnetic radiation spectrum which can be further divided into three diapasons: UV-A (315–400 nm), UV-B (280–315 nm), and UV-C (200–280 nm) [73]. The UV-C range is the most energetic fraction in the UV spectra and can be used to disinfect the surface, water, and food products [74,75]. As the highest germicidal effect is within the range of 250–270 nm, the wavelength of 254 nm is commonly applied in sanitizing surfaces to eliminate most bacteria, viruses, and molds [73,75]. UV-C rays could induce the formation of pyrimidine dimers to alter the DNA helix and block microbial cell replication [76,77,78]. Cells cannot repair the damaged DNA and die [77,79].

Application. The FDA has approved the UV-C light (254 nm) for microbial inactivation in food products, potable water, and juice [80]. It has been proven to reduce foodborne pathogens in fresh produce, and its effectiveness depends on exposure time, irradiation dose, produce type, and pathogen type [81,82,83]. Examples of UV applications are summarized in Table 2. The treatment is more effective on the smooth-skinned produce than on the rough-skinned produce [81]. The UV-C light at 0.92 kJ/m^2^ (60 s) inactivated 2.9 log_10_ CFU/g of *E. coli* O157:H7 on the surface of apple but only 0.5 log_10_ CFU/g on raspberry [81]. The rough surface of produce such as raspberry and cantaloupe can harbor pathogens and be impenetrable to UV-C light [81]. The effectiveness is limited by the shading areas on the produce surface as it provides harbor sites for pathogens. Rotating on conveyance or applying high doses can be an approach to overcome the limitation. However, excess movement and overexposure to UV light may deteriorate the quality of fresh produce. Moreover, pathogen type influences the effectiveness of inactivation. *L. monocytogenes* showed more resistance than *E. coli* O157:H7 under UV treatment [81,84]. The ability to inactivate pathogens also depends on the structure of cells. The thick-walled spores require higher radiation doses compared with the thin-cell bacteria such as *E. coli* [85].

UV-C rays can be used in produce processing and package sanitization before storage to reduce bacterial, yeast, and mold contamination. An increase in the shelf-life of perishable fruits has been demonstrated in recent studies. Moreno et al. (2017) reported that the UV-C treatment of 12.5 kJ/m^2^ reduced the pathogenic and spoilage microorganisms in carambola slices [86]. The treated carambola had a fresh-like appearance after storage for 21 d, while the untreated product showed spoilage and browning symptoms [86]. The oxidation of phenolic compounds such as polyphenol oxidases and peroxidases is the main cause of enzymatic browning and fresh-cut fruit deterioration [87]. The activities of polyphenol oxidases and peroxidases could be inhibited by the UV-C treatment [86].

Combined method. Combining UV-C with other techniques improved the efficiency of pathogen inactivation on fresh produce [82]. For example, combining UV-C with either neutral electrolyzed water or peroxyacetic acid led to about 3 log_10_ CFU/g reduction of *E. coli* and *S. enteritidis* on fresh-cut broccoli, while treated by UV-C only resulted in 1.3–1.4/2.1–2.2 log_10_ CFU/g reduction of *E. coli*/*S. enteritidis*, respectively [88]. UV-C mediated decomposition of ozone and hydrogen peroxide can produce antimicrobial free radicals to inactivate microorganisms on a wide range of fresh produce [89]. A combination of 1 min ozone treatment and 2 min UV-C treatment achieved more than 1–2 log_10_ CFU/g reduction of *E. coli* on blueberry calyx than treated by UV or ozone only [90]. UV-C treatment combined with other technologies to inactivate pathogens on rough surfaces that cannot be reached by UV light may become a new research aspect [81,82,91].

Advantages. UV-C light has been used to sanitize food and non-food contact surfaces, contaminated air, storage freezers, and drinking water [92,93]. It is a non-thermal process and does not involve any harsh chemicals. UV-C lamps are inexpensive and easy to set up. UV-C treatment has minimal effect on the sensory of produce [86,94].

Disadvantages. The limitation is the extremely low penetration level and only effective at surface-level sanitizing [95]. As the UV-C does not penetrate through solids, the dirt and debris on the produce surface could block the UV-C radiation from fully reaching pathogens. The UV-C light poses a potential health hazard depending on the wavelength, intensity, and exposure [96]. It is necessary to shield the user from UV-C light exposure. As overexposure to UV light could cause eye injury, skin burns, and skin cancer, UV-C is considered a health risk to exposed workers [96].

**Table 2:** Examples of UV-based microbial inactivation in fruits and vegetables.

Treatment	Commodity	Treatment Parameters (Dose, Time, Temperature, etc.)	Microorganism	Reduction (log_10_ CFU/g)	Ref.
UV-C light	Apple	0.92 kJ/m^2^, 60 s, 23 °C	*E. coli* O157:H7	2.9	[81]
		3.75 kJ/m^2^, 5 min, 23 °C	*L. monocytogenes*	1.6	[81]
	Pear	0.92 kJ/m^2^, 60 s, 23 °C	*E. coli* O157:H7	2.1	[81]
		11.9 kJ/m^2^, 14 min, 23 °C	*L. monocytogenes*	1.7	[81]
	Strawberry	7.2 kJ/m^2^, 8 min, 23 °C	*E. coli* O157:H7	2.0	[81]
		11.9 kJ/m^2^, 14 min, 23 °C	*L. monocytogenes*	1.0	[81]
	Raspberry	10.5 kJ/m^2^, 12 min, 23 °C	*E. coli* O157:H7	1.1	[81]
	Cantaloupe	11.9 kJ/m^2^, 14 min, 23 °C	*L. monocytogenes*	1.0	[81]
	Fresh-cut carambola	12.5 kJ/m^2^	Aerobic mesophilic bacteria	2.5	[86]
			Yeast and mold	1.9	[86]
	Broccoli	7.5 kJ/m^2^	*E. coli*	1.3–1.4	[88]
			*S. enteritidis*	2.1–2.2	[88]
	Calyx of blueberry	7.95 mW/m^2^, 2 min	*E. coli* O157:H7	1.96	[90]
	Skin of blueberry	7.95 mW/m^2^, 2 min	*E. coli* O157:H7	4.09	[90]
Peroxyacetic acid and UV-C	Broccoli	Peroxyacetic acid, 100 mg/L, pH 5.3, 5 °C + UV-C, 7.5 kJ/m^2^	*E. coli*	~3	[88]
			*S. enteritidis*	~3	[88]
Neutral electrolyzed water and UV-C	Broccoli	Neutral electrolyzed water, 100 mg/L, pH 7, 5 °C + UV-C, 7.5 kJ/m^2^	*E. coli*	~3	[88]
			*S. enteritidis*	~3	[88]
ozone and UV-C	Calyx of blueberry	Ozone 4000 mg/L, 1 min + UV-C 7.95 mW/m^2^, 2 min	*E. coli* O157:H7	3.05	[90]

### 2.3. Cold Plasma

Mechanism. Plasma is a ray of light created by the ionization of gases with sufficient energy such as electricity and heat [97,98]. It consists of gaseous atoms, reactive oxidizing ions, electrons, free radicals, ozone, and UV photons and can be referred to as the fourth state of matter after solid, liquid, and gas [99,100,101,102]. The antimicrobial effect of cold plasma is caused by the chemical reaction with cellular structures and UV damage of DNA and cellular components [100]. As the ionized plasma species can chemically decompose biological and organic molecules and form nonhazardous gaseous such as carbon dioxide and nitrogen dioxide, it has been used in biotechnology, dentistry, cancer and would treatment, sterilization of medical equipment, and food manufacturing [100,103,104].

Application. The non-thermal cold plasma has shown antimicrobial effects on a wide range of microorganisms and opened new applications [100,105]. Cold plasma technology can be designed to generate plasmas inside the package and act as a sanitizing hurdle after the risk elimination of cross-contamination. Examples of cold plasma applications are summarized in Table 3. The *Salmonella*-inoculated tomato and lettuce mixed salads were prepackaged and treated with cold plasma under 35 kV for 3 min inside plastic containers [106]. Results suggested that the efficacy of *Salmonella* spp. inactivation depends on the surface topography of produce, the direction of transfer, and the nature of contamination [106]. The reduction of *Salmonella* spp. was larger on tomatoes than on lettuces as microorganisms had a higher rate of adhering to rough surfaces than on smooth surfaces [106,107]. Pathogen inactivation on smooth surfaces is higher than on rough or irregular surfaces as the irregular surface may shield bacteria from treatment. The efficacy of microbial inactivation increases as surface roughness decreases [102].

Cold plasma has been studied to preserve the qualitative characteristics of produce [98,108]. It reduces the activity of oxidative enzymes such as polyphenol oxidase, and peroxidase, in fresh-cut fruits and vegetables [98,100,109,110]. For example, Ramazzina et al. (2015) utilized the double barrier discharge cold plasma to treat fresh-cut kiwifruit with 10 and 20 min per side and evaluated the quality during 4 days of storage [98]. It was found that cold plasma improved the color retention and reduced the darkened area formation of fresh-cut kiwifruit during storage in the controlled condition and did not induct any textural change [98]. The treatment of cold plasma can positively influence the quality maintenance of kiwifruit with no significant changes in antioxidant content and antioxidant activity [98]. Respiration rate is a critical factor in maintaining the quality of fresh produce and serves as a good indicator of shelf-life [105]. Plasma treatment can alter cellular respiratory pathways and reduce respiration rate to extend storage time [105].

Combined method. In a study by Bhide et al. (2017), *Enterobacter aerogenes* was inoculated onto apple, orange, and cantaloupe peels, which exhibited surface roughness values ranging from 6 to 16 µm. The fruit peels were then treated with cold atmospheric pressure plasma [102]. The apple peel, having the smoothest surface, achieved a 1.25 log_10_ CFU/63.64 cm^2^ higher reduction in bacterial count compared to the cantaloupe peel, which had the roughest surface [102]. Cold plasma-activated hydrogen peroxide aerosol can inactive pathogens on fresh produce [111]. The efficacy of cold plasma-activated hydrogen peroxide depends on the type of produce, the type of bacteria, and the inoculation location of bacteria [112]. For example, after being treated for 45 s with the cold plasma-activated hydrogen peroxide, followed by 30 min dwell time, the populations of *E. coli*, *S. typhimurium*, and *Listeria innocua* were reduced by 4.9, 1.3, and 3.0 log_10_ CFU/piece on the cantaloupe rind, respectively, and 1.5, 4.2, and 4.0 log_10_ CFU/piece on spinach leaves [112]. Jiang et al. (2017) found that reductions of pathogens were higher on the smooth surface of tomatoes than on the stem scar area of tomatoes [112]. The bacteria on the porous area of the stem scar are difficult to inactivate with gaseous antimicrobials [112,113]. Future studies are necessary to investigate the application of cold plasma in combination with various methods for treating produce with rough surface characteristics.

Advantages. Cold plasma is effective in disinfecting fresh produce at atmospheric temperature [100]. It is a surface treatment with minimal impacts on internal nutrients, surface color, and firmness [111,112,114]. Delicate, heat-sensitive, and water-sensitive fresh produce can be effectively treated by cold plasma. It is a low-cost treatment with less maintenance and monitoring [115]. Cold plasma is positive for consumer health with no added chemicals. It extends shelf-life, reduces produce waste, increases the profit of manufacturers and retailers, and provides convenience for consumers [114,116].

Disadvantages. Cold plasma works better on produce that has smooth surfaces, such as apples, compared with the rough surface of cantaloupe [102,106]. The rough and irregular surface of fruits and vegetables such as strawberry and cantaloupe rinds may shield pathogens from cold plasma [102,106]. Additional research is required to understand the potential limits of cold plasm and develop verification and validation experimental guidelines to provide reliable data [100].

**Table 3:** Examples of cold plasma-based microbial inactivation in fruits and vegetables.

Treatment	Commodity	Treatment Parameters (Pulse Frequency, Voltage, Time, etc.)	Microorganism	Reduction (log_10_ CFU/g)	Ref.
Cold plasma	Mixed salad (lettuce inoculated)	35 kV, 3 min	*Salmonella* spp.	0.29	[106]
	Mixed salad (tomato inoculated)	35 kV, 3 min	*Salmonella* spp.	0.54	[106]
	Fresh-cut melon	12.5 kHz, 15 kV, 15 + 15 min	Mesophilic bacteria	1.88	[110]
			Psychrophilic bacteria	0.40	[110]
		12.5 kHz, 15 kV, 30 + 30 min	Psychrophilic bacteria	1.00	[110]
Cold atmospheric pressure plasma	Apple peel	22.5 kHz, 295 V, 492 s, at 199 kPa	*Enterobacter aerogenes*	1.86 ^1^	[102]
	Orange peel	22.5 kHz, 295 V, 492 s, at 199 kPa	*Enterobacter aerogenes*	0.77 ^1^	[102]
	Cantaloupe peel	22.5 kHz, 295 V, 492 s, at 199 kPa	*Enterobacter aerogenes*	0.61 ^1^	[102]
Cold plasma-activated hydrogen peroxide aerosol	Tomato smooth surface	17 kV, H_2_O_2_ 7.8% concentration, 45 s treatment and 30 min dwell time	*S. typhimurium*	5.0 ^2^	[112]
	Tomato stem scar	17 kV, H_2_O_2_ 7.8% concentration, 45 s treatment and 30 min dwell time	*S. typhimurium*	1.3 ^2^	[112]
			*L. innocua*	1.3 ^2^	[112]
					[112]
	Spinach	17 kV, H_2_O_2_ 7.8% concentration, 45 s treatment and 30 min dwell time	*S. typhimurium*	4.2 ^2^	[112]
			*L. innocua*	4.0 ^2^	[112]
			*E. coli* O157:H7	1.5 ^2^	[112]
	Cantaloupe rind	17 kV, H_2_O_2_ 7.8% concentration, 45 s treatment and 30 min dwell time	*S. typhimurium*	1.3 ^2^	[112]
			*L. innocua*	3.0 ^2^	[112]
			*E. coli* O157:H7	4.9 ^2^	[112]

^1^ The unit is log_10_ CFU/63.64 cm^2^; ^2^ The unit is log_10_ CFU/piece.

### 2.4. Pulsed Light

Mechanism. Pulsed light is a non-thermal processing method with ultra-short time and high-peak pulses of broad-spectrum white light including UV (100–400 nm), visible light (400–700 nm), and infrared (700–1100 nm) [117,118,119,120,121,122]. The capacitor accumulates electromagnetic energy during fractions of a second and releases the amplified power in the form of light within nanoseconds or milliseconds [123]. The energy density of pulsed light for food processing applications is about 0.01 to 50 J/cm^2^ at the surface, and the pulses are 1 to 20 flashes per second [123,124,125]. The inactivation function of pulsed light depends on the light between the UV range [126]. The photochemical effect damages the DNA, RNA, and proteins of the microorganisms, while the photothermal effect conveys heat to the surface, leading to their inactivation [126,127]. The pulsed light has a higher instantaneous energy peak and less power consumption than the UV light, leading to effective sterilization [126]. Thus, the pulsed light is four to six times faster than the UV light, inactivating the same amount of microorganisms [127,128,129,130,131]. The FDA concluded that the pulsed light may be safely used for food treatments [132]. The FDA has approved the use of pulsed light technology for the production, processing, and handling of foods, provided the use of xenon flash lamps as the pulsed light source and the cumulative treatment not exceeding 12 J/cm^2^ [133,134].

Application. The intense pulses of short duration have been utilized for the rapid inactivation of microorganisms on the produce surface to extend shelf-life [135,136]. Examples of pulsed light applications are summarized in Table 4. It was reported that the fluences of 2.2 J/cm^2^ reduced 2.3 log_10_ CFU/g of *Saccharomyces cerevisiae* on the tomato surface [137]. However, some appearance defects such as softening, wrinkles, and increased loss of weight were observed after storage for 3 d [137]. Ramos-Villarroel et al. (2011) processed the fresh-cut avocado in plastic trays sealed with polypropylene film for 30 pulses (0.4 J/cm^2^ per pulse) [138]. The reduction of *L. innocua* and *E. coli* after treatment was 2.97 and 3.33 log_10_ CFU/g, respectively [138]. Although pulsed light extended the shelf-life of avocados by the inactivation of microorganisms, browning, softening, and increased respiration rate were observed, affecting the color and firmness of the product [138].

Although the pulsed light is considered a nonthermal method, this is only true when the sample is treated for very short durations [139]. It was reported that a maximum temperature of 64.8 °C was observed on the blueberry during the pulsed light treatment of 60 s [139]. Irregularities with pores on the produce and shadowing effect could harbor the microorganisms from pulsed light [140,141]. The pulsed light system is less effective in lower layers than the upper layer and food pieces may shadow each other in bulk treatment [141]. Also, the high population densities and microorganisms overlap could decrease the inactivation rate [119,141]. For example, Gómez-López et al. (2005) found that the *L. monocytogenes* located on the top were easier to inactivate than those that were covered due to the screen of light by the top-layer bacteria [119].

Combined method. Water-assisted pulsed light processing is a novel method to overcome the shadowing effect and to heat the sample [142]. The *E. coli* O157:H7 inoculated blueberries were immersed in the agitated water and treated by the pulsed light for 5 s [142]. There was a 4.5 log_10_ CFU/g reduction *E. coli* O157:H7 on the skin of blueberries [142]. No viable cells were detected in the wash water, indicating the water-assisted pulsed light effectively prevented cross-contamination during the washing process [142]. No significant differences in color were found between the untreated sample and the treated samples even after 60 s treatment [142]. Water-assisted pulsed light treatment represents a promising non-chemical, residue-free alternative to chlorine washing, offering enhanced antimicrobial efficacy and greater environmental sustainability [142]. Ramos-Villarroel et al. (2015) compared *E. coli* reductions on fresh-cut avocado, watermelon, and mushroom following treatment with pulsed light (180–1100 nm, 12 J/cm^2^) alone and in combination with 2% (*w*/*v*) malic acid [134]. Pulsed light treatment alone resulted in reductions of 2.58, 2.88, and 2.97 log CFU/g on avocado, watermelon, and mushroom, respectively [134]. In contrast, significantly greater reductions were observed when pulsed light was combined with malic acid, achieving 3.14, 3.48, and 3.43 log CFU/g reductions on the same products [134]. Combining pulsed light with organic acids is an effective strategy for enhancing microbial inactivation [134].

Advantages. The pulsed light is a non-thermal technology with low energy cost and non-residual compounds [120]. The treatment process is rapid and amenable to high throughput, as only one to a few flashes are sufficient to inactivate a high level of microorganisms [123]. The pulsed light treatment was found to be four to six times faster than traditional UV light in inactivating the same number of microorganisms [123].

Disadvantages. It is a surface sterilization method for food products and has poor penetration ability [17,126]. The uneven exposure and shadowing effect caused by the rough or irregular surface may block the light and limit the pathogen inactivation [117]. The use of water-assisted or gas-assisted pulsed light systems can help mitigate issues related to shadowing and overheating during treatment. Opaque materials such as polymers and glass are prerequisites if the in-package application of pulsed light is considered [143]. Browning and sample heating may be caused by a long treatment time, leading to the quality deterioration of fresh produce [117,144].

**Table 4:** Examples of pulsed light-based microbial inactivation in fruits and vegetables.

Treatment	Commodity	Treatment Parameters (Dose, Pulse, Time, etc.)	Microorganism	Reduction (log_10_ CFU/g)	Ref.
Pulsed light	Tomato (surface)	2.2 J/cm^2^	*Saccharomyces cerevisiae*	2.3	[137]
	Fresh-cut avocado	0.4 J/cm^2^, 30 pulses	*L. innocua*	2.97	[138]
			*E. coli*	3.33	[138]
	Skin of blueberry	5 J/cm^2^, 5 s	*E. coli* O157:H7	3.8	[142]
		56.1 J/cm^2^, 60 s	*Salmonella* spp.	5.7	[142]
	Fresh-cut avocado	12 J/cm^2^	*E. coli*	2.58	[134]
	Fresh-cut watermelon	12 J/cm^2^	*E. coli*	2.88	[134]
	Fresh-cut mushroom	12 J/cm^2^	*E. coli*	2.97	[134]
Water-assisted pulsed light	Skin of blueberry	5 J/cm^2^, 5 s	*E. coli* O157:H7	4.5	[142]
		56.1 J/cm^2^, 60 s	*Salmonella* spp.	>5.9	[142]
Malic acid and pulsed light	Fresh-cut avocado	12 J/cm^2^, malic acid (2% *w*/*v*), 2 min	*E. coli*	3.14	[134]
	Fresh-cut watermelon	12 J/cm^2^, malic acid (2% *w*/*v*), 2 min	*E. coli*	3.48	[134]
	Fresh-cut mushroom	12 J/cm^2^, malic acid (2% *w*/*v*), 2 min	*E. coli*	3.43	[134]

### 2.5. Ultrasound

Mechanism. Ultrasound is a form of energy generated by sound waves at a frequency higher than 20 kHz, which cannot be heard by the human ear [145,146]. The power ultrasound with low frequency (20–100 kHz) but high energy causes cavitation, which can be utilized in food processing to disinfect microorganisms [145,146,147,148,149,150]. The formation and growth of large resonance-size bubbles (cavitation) can collapse violently and generate high pressure (over 500 bar) locally with high temperatures (up to 5000 °C) [148]. This provides localized mechanical, thermal, and chemical energy to remove dirt and inactivate microorganisms on the product surface [148,151]. The shockwave pressure and shear force are generated when bubbles collapse [151,152,153,154]. Cavitation induces the homolytic cleavage of water molecules, leading to the formation of hydroxy and hydroxyl free radicals (a chemical effect) [148]. Free radicals such as H^+^ and OH^−^ are formed to attack the chemical structure of bacteria cell walls [148]. The temperature of the aqueous medium is an important parameter to maximize cavitation intensity because the viscosity of the medium decreases as the temperature rises [155]. The vapor pressure increases accordingly with temperature increase, allowing the bubbles to develop [155]. However, high to medium temperature leads to an increased volume of vapor inside the bubble, which provides cushion to the collapse and decreases the cavitation intensity [155]. The increased pressure and viscosity hampers cavitation, but the energy release will be greater when implosion occurs [155,156]. The FDA has approved the use of this technology as its ability to achieve a 5-log reduction of foodborne pathogens, which fulfills the microbial safety requirements for fruit juices [157].

Application. Examples of ultrasound applications are summarized in Table 5. Ultrasound treatment was reported to reduce the total aerobic colonies, molds, and yeasts in fresh-cut cucumbers [150]. A 10 min ultrasound treatment effectively reduced weight loss, firmness degradation, and overall color change while also decreasing water mobility and preserving cell wall integrity throughout storage [150].

Combined method. As washing with detergent may not penetrate the crevices on the surface, ultrasonic energy could contribute to the speed and effectiveness of cleaning [158,159]. The ultrasound improves the efficacy of sanitizers such as chlorine, acidic electrolyzed water, sodium dichloroisocyanurate, sodium hypochlorite, peroxyacetic acid, citral, acidified sodium chlorite, and organic acids such as acetic acid, malic acid, citric acid and lactic acid [158,159,160,161,162]. Seymour et al. (2002) reported that the reductions of *S. typhimurium* on iceberg lettuce treated by water, chlorinated water, ultrasound (32–40 kHz) in water, and ultrasound in chlorinated water for 10 min were 0.7, 1.7, 1.5, and 2.7 log_10_ CFU/g, respectively [158]. No *S. typhimurium* was detected in the chlorinated water after washing, while a low level was found in the ultrasound treated water [158]. Chlorine was effective in inactivating bacteria in the wash solution [158]. It is necessary to combine ultrasound with disinfectants to reduce cross-contamination during the washing of fresh produce [158]. It was reported that the population of *E. coli* O157:H7 on the spinach cells was significantly decreased in the samples treated with ultrasound (21.1 kHz) in sanitizing solutions for 2 min at room temperature compared with samples treated with sanitizing solutions alone [160]. The cavity collapse generated high shear and water jets pointing on the produce surface, helping to remove and dislodge bacteria cells [160]. The entrapped bacteria on the surfaces were loosened and displaced by the shearing or scrubbing action [163]. Francisco et al. (2018) reported a 1.46 log_10_ CFU/g reduction of natural microbiota on arugulas after ultrasound (40 kHz) and sodium hypochlorite treatment for 5 min at 25 °C [159]. No significant physicochemical and colorimetric property change was found after the combined action [159].

Nanoemulsion-based encapsulation systems have garnered growing interest, and in a recent study, Song et al. (2024) reported synergistic antibacterial effects against *Shigella flexneri* on fresh-cut carrots when combining the application of ultrasound and a citral nanoemulsion [162]. Thermosonication can be employed to enhance microbial inactivation by combining low-frequency ultrasound waves (around 20 kHz) and mild heat (typically 37–75 °C) [152]. Turhan et al. (2025) investigated the combined effect of thermosonication and organic acids [152]. It was reported that 6.03 log_10_ CFU/cm^2^ reduction in *E. coli* biofilm was achieved using a combined thermosonication and lactic acid treatment at 50 °C for 5 min, while 1.02 log_10_ CFU/cm^2^ reduction was observed with sonication alone at 20 °C for 2 min [152]. The combination of thermosonication and organic acids is a promising hurdle technology for the removal of pathogenic biofilms from fresh produce and food contact surfaces. [152,164].

Advantages. Ultrasound is safe, non-toxic, and considered environmentally friendly [145,154]. The ultrasonic-assisted chemical disinfectant helps to remove microorganisms, biofilm, and particles attached to the surface or hidden in the crevices [146,165]. Minimal loss is found in the flavor, color, and nutritional compounds [154].

Disadvantages. Ultrasound may decrease the texture of peeled fruit as it can activate the degradative polygalacturonases and pectin methylesterase, leading to a soft texture of the tissue [166,167]. The energy requirement and the expense of equipment are relatively high [146]. Treatment parameters such as frequency, time, temperature, water/sample ratio, and agitation-washing protocol are different in each study [145]. Harmonization of results, data comparison, and ultrasound application will be difficult [145].

**Table 5:** Examples of ultrasound-based microbial inactivation in fruits and vegetables.

Treatment	Commodity	Treatment Parameters (Frequency, Time, Temperature, etc.)	Microorganism	Reduction (log_10_ CFU/g)	Ref.
Ultrasound with water	Iceberg lettuce	32–40 kHz, 10 min	*S. typhimurium*	1.5	[158]
	Fresh-cut cucumber	20 kHz, 5 min	Yeast and mold	0.41	[150]
		20 kHz, 15 min	Yeast and mold	0.84	[150]
	Spinach	21.2 kHz, 2 min	*E. coli* O157:H7	2.1	[160]
Ultrasound with PBS	Spinach	35 kHz, 2 min, 20 °C	*E. coli* biofilm	1.02 ^1^	[152]
Ultrasound with chlorinated water	Iceberg lettuce	32–40 kHz, 10 min, chlorinated water (25 ppm)	*S. typhimurium*	2.7	[158]
	Spinach	21.2 kHz, 2 min, chlorinated water (200 mg/L)	*E. coli* O157:H7	3.1	[160]
Ultrasound with sodium chlorite	Spinach	21.2 kHz, 2 min, acidified sodium chlorite (200 mg/L)	*E. coli* O157:H7	4.0	[160]
	Arugula	40 kHz, 5 min, sodium hypochlorite (100 mg/L), 25 °C	Aerobic mesophiles	1.46	[159]
Ultrasound with peroxyacetic acid	Spinach	21.2 kHz, 2 min, peroxyacetic acid (80 mg/L)	*E. coli* O157:H7	2.9	[160]
Ultrasound with acidic electrolyzed water	Spinach	21.2 kHz, 2 min, acidic electrolyzed water (80 mg/L)	*E. coli* O157:H7	3.1	[160]
Ultrasound with citral nanoemulsion	Fresh-cut carrot	20 kHz, 9 min, citral nanoemulsion (0.15 mg/mL)	*Shigella flexneri*	8.55 ^2^	[162]
Thermosonication	Spinach	35 kHz, 5 min, 50 °C	*E. coli* biofilm	4.19 ^1^	[152]
Thermosonication with acetic acid	Spinach	35 kHz, 5 min, acetic acid (20 mL/L), 50 °C	*E. coli* biofilm	5.94 ^1^	[152]
Thermosonication with lactic acid	Spinach	35 kHz, 5 min, lactic acid (20 mL/L), 50 °C	*E. coli* biofilm	6.03 ^1^	[152]

^1^ The unit is log_10_ CFU/cm^2^; ^2^ The unit is log_10_ CFU/mL.

### 2.6. High-Pressure Processing

Mechanism. High-pressure processing, also described as high hydrostatic pressure, is a non-thermal processing technique aiming to maintain the natural and fresh quality of food [139]. The food is packaged in a flexible pouch or plastic container and loaded in an opening basket, which is then introduced into the pressure vessel. The vessel is filled with liquid, typically water, to convey pressure [139]. The pressure is increased to the target pressure, which is usually between 100 and 1000 MPa at room temperature [75,168]. The pressure is transmitted throughout the product uniformly and simultaneously [169]. The high isostatic pressure can cause changes in microorganisms in the morphology, cell membrane, and biochemical reactions [170]. It mainly affects noncovalent bonds, while thermal processing destroys covalent bonds [171]. The high pressure alters the high molecular weight proteins and carbohydrates through rupturing their noncovalent bonds [139]. The cell wall and membrane of microorganisms are disrupted, and enzymes are inactivated [171,172,173]. The flavoring agents, pigments, and vitamins are less affected by high-pressure processing since they are low molecule-weight compounds and contain covalent bonds [139,171,173]. As there are minimal chemical changes in the high-pressure processed food, the sensory properties are maintained [139,171,173].

Application. The FDA and USDA have approved this technology for application in food processing, opening opportunities for new markets. It has been applied in processing juice, smoothies, baby food, plant-based protein drinks, broth, coffee, ready-to-eat meat, soups, sauce, seafood, fruits, and vegetables. Generally, the vegetative bacteria, yeast, and mold can be inactivated at a pressure above 200 MPa, and bacterial spores may be inactivated at a pressure above 1000 MPa [174]. Examples of high-pressure processing applications are summarized in Table 6. It was reported that exposure to 200 MPa resulted in approximately a 1-log reduction in PCA counts across all tested temperature (4, 21, and 38 °C) and time (5, 15, 40, and 60 min) combinations on fresh-cut pineapple [175]. At 270 MPa for 15 min, reductions greater than 2 logs were observed at 38 °C [175]. The quality and storage stability of green beans preserved by high-pressure processing (60 s, 500 MPa, 20 °C) were evaluated [176]. After one month of storage, no significant microbial outgrowth was observed [176]. Compared to conventional preservation methods, the high-pressure treatment resulted in significantly better retention of firmness and ascorbic acid content [176]. The natural presence of such oxidoreductase enzymes in fruits and vegetables could influence the nutrition and quality by interaction with phenolic compounds and the production of brown-colored complexes [177,178]. The high-pressure processing can inactivate quality-deteriorating enzymes to maintain color, flavor, texture, and nutritional values [177,178].

Combined method. Vercammen et al. (2012) demonstrated the inactivation of *Candida lipolytica* and *E. coli* on 1 cm apple cubes immersed in acidified glucose solutions (12.5% or 25.0% D-glucose) using high-pressure treatment [179]. At 25 °C, a 6-log reduction of *C. lipolytical* and *E. coli* was observed at 400 MPa and 600 MPa for 10 min, respectively, across both tested solutions [179]. Tola and Ramaswamy (2015) compared high-pressure-assisted thermal processing with traditional thermal treatment in citric acid-infused carrots [180]. The texture retention was higher in high-pressure-assisted thermal processed carrots than those processed under conventional canning [180]. However, there was no difference in color change between the two methods [180]. High-pressure processing is widely used for juices and purees. Further research is needed to explore its combined use with other methods for enhancing microbial inactivation in fresh and fresh-cut fruits and vegetables.

Advantages. High-pressure processing can effectively control pathogens and spoilage microorganisms [181]. It maintains food taste, texture, and nutritional quality as products are exposed to minimal processing without heat [177,181]. It extends shelf-life to reduce food waste [139]. As the fresh product does not contain chemical additives and preservatives, it supports a clean label [182]. High-pressure processing can use recyclable water, which is environmentally friendly [182].

Disadvantages. The cost of high-pressure processing is relatively high. The packaging material should be water-resistant and flexible to withstand compression [183,184]. The cell structure of delicate fruits and vegetables may not withstand the pressure and lead to irreversible damage.

### 2.7. Microbubbles and Nanobubbles

Mechanism. Water bubbles are usually categorized into three types: ordinary macrobubbles (100 μm–2 mm in diameter), microbubbles (1–100 μm in diameter), and nanobubbles (less than 1 μm in diameter) [185,186]. The ordinary marcobubble in the liquid can rise to the surface quickly and collapse [186]. The microbubble shrinks in the liquid medium and then dissolves into it [186,187]. The nanobubble is stable and can stay in the solutions longer for its negative surface charge, low buoyancy, and Brownian motion [186,188,189].

Microbubbles have been utilized in the washing process [190]. The shock wave, shear, and lift forces from the bubbles collapsing can remove bacteria and dirt particles from the surface [191,192]. Nanobubbles are also a novel approach to removing pathogens, biofilm, and organic material from the surface [193,194]. Biofilms have high resistance to sanitizer and can persist in the environment for a long time [195]. Nanobubbles can reduce the surface tension and pathogen adhesion on the contact surface [195].

Application. Examples of microbubble and nanobubble applications are summarized in Table 7. Washing *S. typhimurium* inoculated sweet basil with microbubble water for 7 min at 30 °C resulted in 1.3–1.8 log_10_ CFU/g reduction [190]. However, a concentration of 2.77 log_10_ CFU/mL was detected in the microbubble water, which may increase the risk of cross-contamination [190]. Although washing with microbubbles alone may not result in large bacteria reduction, combining microbubbles with other technologies such as ozone and sanitizers is a new method for the fresh produce industry to effectively remove and inactivate microorganisms [190,196].

Combined method. Washing *S. typhimurium* inoculated sweet basil with ozone microbubble water (1 mg O_3_/L) led to 2.6 log_10_ CFU/g reduction [190]. No survival of *S. typhimurium* was found in the ozone microbubble water after washing [190]. Cross-contamination was prevented in the ozone microbubbles [190]. Surface roughness influences bacterial cell detachment, with pathogens being more easily removed from smoother surfaces [196]. Chuajedton et al. (2016) processed fresh-cut pineapple with ozone microbubbles at different ozone concentrations and treatment time intervals. It was concluded that the ozone microbubbles could potentially extend the shelf-life [197]. Rafeeq and Ovissipour (2021) combined the nanobubble and ultrasound to remove *L. innocula* and *E. coli* O157:H7 on the spinach surface, and this resulted in more than 2 and 4 log_10_ CFU/cm^2^, respectively [193]. *L. innocula* was more resistant than *E. coli* O157: H7 in the treatment [193].

Advantages. The microbubble and nanobubble are small in size and have large specific surface areas [198]. It can reside in the water for a long time [198]. The application of microbubble and nanobubble technologies in fresh produce washing is an emerging trend among researchers. Microbubbles and nanobubbles can be utilized to remove bacteria from surfaces [190,195,199]. Additional sanitizers or methods are added to inactivate pathogens and prevent cross-contamination of foodborne illnesses [199]. It is a sustainable solution for managing the postharvest quality and safety of fresh produce [200].

Disadvantages. The generation of microbubbles and nanobubbles requires relatively high energy consumption, which can pose challenges for large-scale production [200]. Additionally, the low bubble density limits scalability [200].

### 2.8. Electrolyzed Water

Mechanism. Electrolyzed water is generated by electricity applied to water with salt content such as NaCl and HCl [172,201,202,203,204]. The efficacy of electrolyzed water on microbial inactivation depends on various parameters such as the available chlorine concentration, oxidative reduction potential, organic matter in the water, temperature, pH, agitation, and water hardness [172,205]. The oxidant sanitizer that produces free chlorine contains active Cl_2_, HOCl, and OCl^−^ [205]. The presence of ions and the high oxidative reduction potential of electrolyzed water can inactivate microorganisms by damaging the cell membranes and disrupting the metabolism inside the cells [172]. Organic matter reduces the amount of free chlorine and decreases the sanitizing ability of electrolyzed water [205,206]. Chlorine is most effective in the HOCl form when the solution pH is between 5.0 and 6.5 [205,207]. Increasing the temperature of the solution leads to an increased sanitizing efficacy [205,207].

Application. Washing produce with sodium hypochlorite solution is a common method in minimally processed fruit production but has environmental and safety issues [208]. The electrolyzed water has antimicrobial activity against pathogens in food products and processing surfaces [209]. Thus, the electrolyzed water is a good alternative to sodium hypochlorite [204,210]. Examples of electrolyzed water applications are summarized in Table 8. Lopes et al. (2021) found that processing fresh-cut mango with neutral electrolyzed water (150 mg free active chlorine/L) at 10 °C for 15 min resulted in below 3.0 MPN 100/g (below the detection limit) of thermotolerant coliforms [203]. The nutritional components and sensory aspects of the electrolyzed water-treated mango were preserved [203]. Ding et al. (2024) reported that the use of acid-electrolytic water can help improve the quality of fresh-cut red pitaya fruit [211].

Combined method. The electrolyzed water can be combined with chemical sanitizers such as calcium oxide and fumaric acid or physical treatments such as microbubbles, ultrasound, and ultraviolet irradiation to improve the sanitizing effect [163,204,212]. Tango et al. (2017) washed the *E. coli* O157:H7 and *L. monocytogenes* inoculated apple and tomato with different chemical and physical methods [212]. They found that washing with calcium oxide for 3 min, followed by washing with a mixture of fumaric acid and slightly acidic electrolyzed water in the ultrasound bath for 3 min, led to a high microbial reduction (more than 5 Log_10_ CFU/fruit) [212]. Ngnitcho et al. (2017) reported that a combination of electrolyzed water with calcium oxide and fumaric acid was a favorable method to inactivate pathogens and extend the shelf-life of produce such as spinach, iceberg lettuce, and alfalfa sprouts, with little quality damage [212]. Significant synergistic effects can be achieved through combined treatments for the effective elimination of bacterial pathogens from fresh produce [212].

Advantages. Electrolyzed water has gained immense popularity as a broad-spectrum sanitizer [205]. It is simple to produce and has low operational costs [213]. It uses cheap raw materials and has low trihalomethane generation [204]. The food quality is minimally influenced by the electrolyzed water [205,211].

Disadvantages. The strong acid-electrolyzed water has a high inactivation rate but may have corrosive problems [172,214]. Chlorine gas may be released during the operation of an electrolyzed water system [205].

### 2.9. Chlorine Dioxide Gas

Mechanism. Chlorine dioxide is internationally recognized as an emerging antimicrobial agent and preservative, and its use has been widely adopted across various industries and countries [215]. Chlorine dioxide is a strong oxidizing agent which has an oxidation capacity of about 2.5 times of chlorine [15,216]. The chlorine dioxide gas has antibacterial ability through the destabilization of cell membrane, alteration of the permeability of cell membrane, disruption of cell metabolism, and interruption of protein synthesis [217]. Chlorine dioxide gas can be generated by the reaction of an acid with sodium chlorite, sodium chlorate, or chlorine gas [217,218,219]. The FDA has approved chlorine dioxide gas for produce processing [220].

Application. The pathogen can penetrate deeply if the produce surface is injured, leading to a challenge to decontamination with liquid wash as compared to gaseous treatment such as chlorine dioxide gas [221]. The chlorine dioxide gas has great potential for pathogen inactivation, as the gas can enter into wounds and stem scars where pathogens were harbored. Examples of chlorine dioxide gas applications are summarized in Table 9. Han et al. (2001) processed the *L. monocytogenes* inoculated green peppers with gaseous chlorine dioxide (3 mg/liter) and aqueous chlorine dioxide (3 mg/litter) [221]. There were 7.39 and 3.60 log_10_ CFU/5 g reduction from uninjured surface and injured surface under gaseous treatment, while only 3.67 and 0.44 log_10_ CFU/5 g reduction under aqueous treatment [221]. Gaseous chlorine dioxide was more effective in inactivating *L. monocytogenes* on both uninjured and injured green peppers than chlorine dioxide solution [221]. Mahovic et al. (2007) distributed the chlorine dioxide gas up to 99 mg over the wound-inoculated tomato fruit in a sealed container and found that the wound remained firm and dry after 24 h [222]. Meanwhile, the control tomato without chlorine dioxide treatment showed bacterial activity and soft rot [222]. It was concluded that the chlorine dioxide gas may be effective in controlling the postharvest decays of produce [222]. Chlorine dioxide gas residues on a wide variety of produce (lettuce, cantaloupe, tomato, apple, strawberry, orange, and alfalfa sprouts) have been studied [223]. Trinetta et al. (2011) treated the produce with different combinations of concentrations of chlorine dioxide gas and time and monitored the residues on the surface. They detected very low residues in most of the produce, except for lettuce and alfalfa sprouts which showed relatively higher residues [223]. It was concluded that the chlorine dioxide gas had minimal to no detectable residues in some produce, indicating no significant risk to people [223].

Combined method. The antimicrobial effect of the combined treatment of UV-C and chlorine dioxide gas was evaluated on tomatoes [224]. After 20 min treatment of UV-C and chlorine dioxide gas (10 ppmv), there were 4.80, 4.28, and 2.7 log_10_ CFU/g reductions for *E. coli* O157:H7, *S. typhimurium*, and *L. monocytogenes*, respectively [224]. The food quality of spinach leaves was maintained for 7 days during storage [224]. Combining chlorine dioxide gas with other methods can be effectively applied for the inactivation of foodborne pathogens on fresh produce, including during its transportation and storage [224].

Advantages. Chlorine dioxide gas is effective over a wide range of pH [215]. The gas can cover produce surfaces and enter into wounds and stem scars to inactive pathogens [15,221]. It does not produce toxic by-products nor alter nutrition and organoleptic quality [218]. It is effective against biofilm [225]. Chlorine dioxide gas exhibits a strong antimicrobial effect while exerting minimal environmental impact [226]. It can be applied to the package directly [226].

Disadvantages. The chlorine dioxide gas should be prepared onsite before use, as the gas cannot be compressed nor transported under pressure [218,227]. Chlorine dioxide gas may interact with package materials or permeate through the package material [228]. The selection of an appropriate barrier material is necessary.

## 3. Conclusions

The growing consumer demand for fresh, nutritious, and microbiologically safe produce has driven significant interest in emerging and innovative sanitization technologies. This review evaluated a range of novel technologies, highlighting their mechanisms of action, efficacy against foodborne pathogens, and potential applications in fresh produce processing. No single technology is universally applicable across all types of produce and contamination scenarios. Ozone and chlorine dioxide gas exhibit strong oxidative properties and can be applied to disinfect produce with rough or irregular surfaces. Ultraviolet light and pulsed light are more effective on smooth-surfaced fruits and vegetables and can combine with other technologies to enhance their antimicrobial efficacy. High-pressure processing is appropriate for produce that is not delicate and can tolerate compression while effectively preserving nutritional quality. Ultrasound, electrolyzed water, microbubbles, and nanobubbles demonstrate improved microbial inactivation when used in combination with sanitizing agents.

Integrated approaches that combine multiple treatments may offer synergistic benefits, optimizing microbial reduction while maintaining sensory and nutritional quality. The use of hurdle technology, where multiple mild interventions are combined to achieve enhanced efficacy, is gaining traction in fresh produce sanitation. Additionally, bioactive compounds such as plant-derived antimicrobials and essential oils are being explored as natural and residue-free alternatives to synthetic sanitizers. Cold plasma, with its ability to generate reactive species at low temperatures, is emerging as a promising non-thermal intervention with broad-spectrum antimicrobial potential.

As research continues to advance, greater emphasis should be placed on developing economically viable, environmentally sustainable, and consumer-acceptable sanitization solutions. Additionally, standardization in evaluation protocols will be crucial to enable meaningful comparison across technologies and ensure reproducibility of results. While these technologies have good potential for enhancing food safety with minimal impact on produce quality, their practical implementation at industrial scales remains complex. Factors such as energy consumption, cost-effectiveness, regulatory acceptance, and compatibility with different produce types influence their scalability and broader adoption.

## Figures and Tables

**Table 6:** Examples of high-pressure processing-based microbial inactivation in fruits and vegetables.

Treatment	Commodity	Treatment Parameters (Pressure, Time, Temperature, etc.)	Microorganism	Reduction (log_10_ CFU/g)	Ref.
High-pressure processing (HPP)	Fresh-cut pineapple	270 MPa, 15 min, 38 °C	Viable bacteria on PCA	>2	[175]
		340 MPa, 15 min, 4 °C	Viable bacteria on PCA	3.0	[175]
	Green bean	500 MPa, 60 s, 20 °C	Vegetative bacteria	4.4	[176]
		500 MPa, 60 s, 20 °C	Spore	1.8	[176]
HPP and acidified glucose solutions	Apple cube	400 MPa, 10 min, 25 °C, acidified glucose solution (12.5 or 25.0% D-glucose)	*C. lipolytica*	~6	[179]
			*E. coli*	~6	[179]
		600 MPa, 10 min, 25 °C, acidified glucose solution (12.5 or 25.0% D-glucose)	*C. lipolytica*	~6	[179]
			*E. coli*	~6	[179]

**Table 7:** Examples of microbubble and nanobubble-based microbial inactivation in fruits and vegetables.

Treatment	Commodity	Treatment Parameters (Bubble Size, Time, Temperature, etc.)	Microorganism	Reduction (log_10_ CFU/g)	Ref.
Microbubble	Sweet basil	1.23–3.41 μm, 7 min, 30 °C	*S. typhimurium*	1.3–1.8	[190]
Microbubble and ozone	Sweet basil	1.23–3.41 μm, 7 min, 30 °C, ozone (1.0 mg/L)	*S. typhimurium*	2.6	[190]
Microbubble and ozone	Sweet basil	1.23–3.41 μm, 7 min, 10 °C, ozone (2.0 mg/L)	*S. typhimurium*	2.2	[190]
Nanobubble and ultrasound	Spinach	20 min, Ultrasound (40 Hz)	*L. innocua*	>2 ^1^	[193]
			*E. coli* O157:H7	>4 ^1^	[193]

^1^ The unit is log_10_ CFU/cm^2^.

**Table 8:** Examples of electrolyzed water-based microbial inactivation in fruits and vegetables.

Treatment	Commodity	Treatment Parameters (Concentration, Time, Temperature, etc.)	Microorganism	Results	Ref.
Electrolyzed water	Fresh-cut mango	150 mg free active chlorine/L	Coliform	<3.0 MPN 100/g ^1^	[203]
			Psychrotrophic bacteria	<10 CFU/g ^1^	[203]
			Yeast and mold	<10 CFU/g ^1^	[203]
Slightly acidic electrolyzed water	Apple	3 min, 23 °C	*E. coli* O157:H7	2.28 log_10_ CFU/fruit ^2^	[212]
			*L. monocytogenes*	2.30 log_10_ CFU/fruit ^2^	[212]
	Tomato	3 min, 23 °C	*E. coli* O157:H7	2.73 log_10_ CFU/fruit ^2^	[212]
			*L. monocytogenes*	2.35 log_10_ CFU/fruit ^2^	[212]
Calcium oxide, fumaric acid, slightly acidic electrolyzed water, ultrasound	Apple	Calcium oxide (3 min) + mixture of fumaric acid and slightly acidic electrolyzed water in the ultrasound bath (40 Hz), 3 min, 23 °C	*E. coli* O157:H7	>5 log_10_ CFU/fruit ^2^	[212]
			*L. monocytogenes*	>5 log_10_ CFU/fruit ^2^	[212]
	Tomato	Calcium oxide (3 min) + mixture of fumaric acid and slightly acidic electrolyzed water in the ultrasound bath (40 Hz), 3 min, 23 °C	*E. coli* O157:H7	>5 log_10_ CFU/fruit ^2^	[212]
			*L. monocytogenes*	>5 log CFU/fruit ^2^	[212]

^1^ Survival after treatment; ^2^ Reduction after treatment.

**Table 9:** Examples of chlorine dioxide gas-based microbial inactivation in fruits and vegetables.

Treatment	Commodity	Treatment Parameters (Concentration, Time, Temperature, etc.)	Microorganism	Reduction (log_10_ CFU/g)	Ref.
Gaseous chlorine dioxide	Green pepper (uninjured surface)	3 mg/L, 10 min, 20 °C	*L. monocytogenes*	7.39 ^1^	[221]
	Green pepper (injured surface)	3 mg/L, 10 min, 20 °C	*L. monocytogenes*	3.60 ^1^	[221]
	Spinach	10 ppmv, 20 min	*E. coli* O157:H7	3.56	[224]
			*S. typhimurium*	3.61	[224]
			*L. monocytogenes*	3.23	[224]
	Tomato	5 ppmv, 20 min	*E. coli* O157:H7	2.34	[224]
			*S. typhimurium*	2.24	[224]
			*L. monocytogenes*	1.57	[224]
UV-C and chlorine dioxide gas	Spinach	10 ppmv, UV-C (70.68 μW/cm^2^), 20 min	*E. coli* O157:H7	5.17	[224]
			*S. typhimurium*	5.47	[224]
			*L. monocytogenes*	4.32	[224]
	Tomato	5 ppmv, UV-C (70.68 μW/cm^2^), 20 min	*E. coli* O157:H7	4.80	[224]
			*S. typhimurium*	4.28	[224]
			*L. monocytogenes*	2.70	[224]

^1^ The unit is log_10_ CFU/5 g green pepper.

## Data Availability

No new data were created or analyzed in this study. Data sharing is not applicable to this article.
